# “Someone Should be There to Take Care of It”: A Case Study of Users’ Views of Managed Shared Sanitation Facilities in Jharkhand, India

**DOI:** 10.4269/ajtmh.21-0654

**Published:** 2022-02-21

**Authors:** Sharon Vu, Anoop Jain, Caleb Harrison, Prabin Ghimire, Jay P. Graham

**Affiliations:** ^1^ University of California–Berkeley, Berkeley, California;; ^2^ Global Health & Social Medicine, Harvard Medical School, Boston, Massachusetts;; ^3^ University of Michigan School of Public Health, Ann Arbor, Michigan;; ^4^ Sanitation and Health Rights in India Cooperative Colony, Bokaro Steel City, Jharkhand, India;; ^5^ School of Public Health, Berkeley, California

## Abstract

The Sustainable Development Goals have set an ambitious target to end open defecation by 2030 by building private household toilets. These toilets are categorized based on quality indicators. However, toilets that are shared among households are considered “limited,” disincentivizing governments and implementers from investing in this infrastructure despite being more appropriate in certain contexts. Furthermore, unlike private toilets, shared toilets are not distinguished based on their quality. As such, there is a need to understand what attributes constitute well-managed shared toilets. These types of facilities could play an important role in helping people move up the sanitation ladder away from open defecation in certain contexts. Therefore, we conducted 41 one-on-one in-depth interviews with users of managed shared sanitation facilities. We found that maintenance and accessibility are key indicators of well-managed shared sanitation. Maintenance includes the provision of water for flushing and self-cleaning, cleaning, and high-quality built infrastructure. Accessibility is defined by the distance people have to walk to reach the facility, the amount of time they have to wait in line, and design features of the facility that encourage use. These findings could help distinguish managed versus unmanaged shared sanitation and could help inform global sanitation policies.

## INTRODUCTION

Governments, policymakers, and implementers continue prioritizing the construction of private household toilets in an effort to achieve the ambitious target set by Sustainable Development Goal (SDG) 6.2 of eliminating open defecation by 2030. ^1^ To ensure a certain minimum quality of these private facilities, the WHO’s Joint Monitoring Program (JMP) has categorized these toilets according to certain attributes. Private toilets are “safely managed” (the highest quality) if they are improved—flush/pour flush toilets connected to piped sewer systems, septic tanks, or pit latrines; pit latrines with slabs, and composting toilets—and if the excreta are safely disposed of in situ or removed and treated offsite. ^1^ Private toilets that are improved but where the excreta are not safely treated are considered “basic.” Finally, pit latrines without a slab, hanging latrines, and bucket latrines are all considered “unimproved.”

These definitions and categorizations have created an incentive for governments to build “safely managed” toilets so that they can show progress toward meeting the SDG’s sanitation target by 2030. The Indian government, for example, launched its own national sanitation program called Swachh Bharat Abhiyan (Clean India Mission) in October 2014. ^2^ This government program incentivizes the construction of “safely managed” household toilets where participants can receive up to 12,000 Indian Rupees upon successful completion of their in-home toilet. ^3^ As a result of these efforts, the percentage of people defecating in the open decreased from 29% in 2015 to 15% in 2020, and the number of people using “safely managed” toilets rose from 36% in 2015 to 46% in 2020. ^1^

Not all improved toilets count toward SDG 6.2. More specifically, improved toilets that are shared among households are considered “limited.” ^1^ This distinction between private and shared toilets creates a disparity between those that have a toilet and those that do not. ^4^ As a result, the improved shared facilities used by more than 165 million people throughout India do not count toward SDG 6.2. The distinction between private and shared toilets also disincentivizes governments, researchers, and implementers from examining what constitutes managed shared sanitation, how it can be implemented, and under what circumstances. ^4^

However, the JMP does not distinguish between managed (sanitation blocks that have operations and maintenance service support) and unmanaged shared sanitation. Private toilets, compared with unmanaged shared toilets, might be more effective at ensuring better health, safety for women, and access to water. ^5^^–^ ^10^ Yet managed shared facilities could play an important role in helping eliminate open defecation while preventing adverse outcomes. One study, for example, showed no increased risk in trachoma between those who use shared versus private toilets. ^11^ In some cases, increased access to shared facilities reduced diarrheal and gastrointestinal diseases. ^12^ Another study showed that although private toilet ownership did help reduce the risk of nonmarital sexual violence in rural areas, this was not the case in urban settings, ^13^ where shared sanitation might be most useful due to high population densities. Furthermore, shared sanitation facilities have been found to reduce women’s feelings of stress due to fear of violence if they are managed and well maintained. ^14^ Interventions to ensure access to water for flushing, self-cleaning, and menstrual hygiene management have also been effective at encouraging shared toilet use in Dhaka, Bangladesh. ^15^ Thus, managed facilities might be wholly different from unmanaged shared facilities in the same way that improved and unimproved private toilets are inherently different.

Moreover, not recognizing a role for managed shared sanitation in certain contexts potentially limits safe, interim sanitation options for households that face structural barriers to private toilet ownership. Many parts of the country are unlikely to address structural barriers, including prohibitive cost of private toilet construction, the lack of space for a toilet, and inadequate water supply, essential for self-cleaning and flushing (pit latrines), in the short term, and well-managed shared sanitation options are likely a critical step forward in specific contexts. ^16^^–^ ^20^ Furthermore, 82% of India’s population in 2011 lived on land that they did not own. ^21^ Uncertain land tenure discourages investment in built infrastructure, such as toilets, because it may be removed by landowners. ^22^^,^ ^23^

This underscores the need for additional research that examines local voices and user experiences pertaining to what constitutes managed shared sanitation. These voices remain conspicuously absent from the process of defining global sanitation standards such as those set by the JMP. To “elicit the experiences and views of poor people and to incorporate these views into all observations, judgments, and actions” is an essential step toward achieving equity-based global health goals such as the SDGs. ^24^ In the context of managed shared sanitation, user experiences are required to elucidate the attributes of these facilities, ^22^ thereby dismantling the dichotomy between safely managed facilities and everything else that in many cases acts as a “straight-jacket that alienates worthwhile efforts.” ^4^

Therefore, the purpose of this study was to understand user perspectives on the essential attributes of managed shared sanitation facilities and what role these facilities can play in people’s lives. Understanding these perspectives is particularly important in India because managed shared toilets could help more than 200 million people who defecate in the open move up the sanitation ladder while ensuring high-quality service provision. ^1^ To this end, we conducted structured interviews with 41 individuals in the Bokaro district of Jharkhand, a state in India, that use managed shared sanitation facilities. This study is significant because it fills a critical gap in the literature by giving a voice to managed shared sanitation users. ^22^^,^ ^25^ These perspectives could help inform future research pertaining to shared sanitation that could in turn shape global sanitation policies regarding the role of shared sanitation infrastructure.

## METHODS

We conducted a qualitative case study in two informal settlements in Bokaro, Steel City, a large industrial city in the Bokaro District of Jharkhand, India. The two informal settlements, Dundhi Baag and Basgoda, offered unique perspectives into the needs of residents of informal settlements and how managed shared sanitation plays a role in meeting those needs. Dundhi Baag, which is about 0.3 km^2^ in size and sits adjacent to a local outdoor market, received a managed shared sanitation facility in 2017. At the time of this study, Basgoda, a similarly sized informal community located nearby, was in the process of constructing a similar shared sanitation facility. We chose these sites because one allowed us to gain the perspective of current users of the facility, and the other helped us gather input for the facility under construction.

We conducted semistructured, one-on-one interviews with individuals to understand their sanitation needs, past and present barriers to sanitation access, and their lived experiences defecating in the open and using shared facilities. We interviewed a total of 41 adults: 25 from Dundhi Baag and 16 from Basgoda. We used convenience sampling for selecting study participants; however, selection criteria (i.e., age, sex, household latrine ownership status, and proximity to the facility) were used to identify subgroups who could provide different perspectives. The University of Michigan Institutional Review Board reviewed and approved the study design (Study protocol HUM00161735).

### Data collection and analysis.

Interviews took place between June and July 2019. Translation and transcription services (https://fieldscope.in/) were used to translate and transcribe all interviews. Interviews were conducted in Hindi by two lead researchers and recorded and transcribed. Translations of the transcribed interviews to English were carried out by a professional translator. All participants gave informed verbal consent to participate in this study.

Thematic analysis was used to identify the themes from interviews through a multistep process. Specifically, two researchers individually coded each transcript line-by-line using ATLAS.ti (version 8.3.1, www.atlasti.com) to iteratively develop the first draft of the codebook, which was then reviewed for intercoder reliability. Codes were then grouped into broader themes based on patterns we collaboratively discussed, with a strong focus on themes related to 1) peoples’ perception of their managed shared sanitation facility, 2) barriers to private toilet ownership, 3) reasons why OD persists, 4) how OD makes participants feel, and 5) government’s role in the sanitation discourse. These themes are discussed next.

## RESULTS

### Defining maintenance.

The majority of study participants described how managed shared sanitation facilities should be well-maintained. Overall, they spoke about three key dimensions of maintenance. These were the provision of water, professional management of the facility, and the quality of the built infrastructure.

Study participants reported facing extreme water shortages where they live. The dearth of water, for drinking, household chores, and sanitation use, was described as an immutable characteristic of the community. Many wells have dried up, and water is “only available very far below the ground.” Study participants stated that to have a hand pump for water at home, they would have to hire a special machine that would be able to “dig up to 650 feet deep to find water.” As a result, many of the participants relied on a government water pump that was inconveniently located. People struggled because “they could not clean themselves or flush after using the toilet” as water was not always readily available at home. Many who once had a private toilet would “instead have to defecate at the river.” There was only one hand pump in the vicinity that “gets really crowded because everyone goes there,” which further discouraged people from bringing water to their home for their sanitary needs.

As such, study participants suggested that ensuring an adequate water supply be an essential part of a shared sanitation facility maintenance checklist. They even noted that people would not use the facility “if water was not available.” Having water is essential for flushing and self-cleaning as a key indicator of shared sanitation facility quality. Beyond flushing and self-cleaning, the participants stated that having a water supply available at a shared sanitation facility was essential for cleaning. This is particularly important when it rains. In addition to a dearth of water, there is a dearth of paved roads in the communities where our participants live. As a result, the “roads become very muddy” when it rains, filling the facility with “mud and dirt as people come and go.” Therefore, shared sanitation facility maintenance should ensure an adequate supply of water for flushing, self-cleaning, and ensuring facility cleanliness.

Study participants consistently expressed that shared sanitation facilities should be professionally operated. They stressed the importance of having paid staff that would be responsible for facility maintenance. One person said that if a facility is built, “someone should be there to take care of it.” Another said, “If it is clean, people will go there 24 hours a day.” People also wanted to make sure that the facility was not contributing to the pollution of their community. It should be managed in such a way that the facility is “connected to a sewer line so that the waste does not flow into the river.” This was important given that many people still use the river for bathing and household chores. Some participants were adamant that a portion of this responsibility—of keeping a facility maintained—rests with the community members themselves. One man said that the quality of the sanitation facility “depends on how the public keeps it.” He said, “We have to keep it clean ourselves” and that people should not leave it dirty and leave. People insisted that the public needed to play their role in ensuring facility maintenance. One participant said, “People need to use water to clean the toilet after using it, that’s how it will stay clean”.

In some extreme cases, the lack of maintenance forced facility closures, forcing participants to revert to open defecation. Participants remembered a pay-for-use shared facility that used to operate nearby. The facility operators “charged five Rupees per use.” Despite this, “it stopped working because there was no maintenance,” which ultimately led to it being shut down. Our participants indicated that they would be happy to pay for toilet use so long as the facility was “being cleaned and washed.” One woman recalled how people stopped going because it became very dirty and that some people were even afraid that “the roof might fall down.” Therefore, in addition to being kept clean, our participants believed that for a shared sanitation facility to be of high quality, it also had to be structurally sound and aesthetically appealing. This result is summarized in Table 1.

### The many dimensions of access.

The availability of a shared sanitation facility was an interim solution while our participants waited patiently for structural changes that would allow them to build a private household toilet. Uncertain land rights, inadequate dwelling space, and poverty were some of the barriers that our participants faced when trying to build a private household toilet for their families. For example, they spoke candidly about their tenuous financial situations as a major constraint. Participants noted that households in this community “have five to six family members to feed but only earn 200 to 300 rupees per day,” a clear indication that building a private toilet was infeasible. That’s why one participant acknowledged that the shared facility was “good for everyone”, especially those who could not afford their own private toilet, and those who did not have space for one. Although many said that “there should be a toilet at home,” they understood that a shared facility might be their best option as they “live day to day.” The shared facility also assuaged people’s fears about having to manage waste because they no longer had to worry about their pits filling up. In that sense, some even likened the shared facility to a centralized municipal waste management facility seen “in big cities” that use “pipes and drains” to manage the waste. They knew that this could help them save around “2,000 to 5,000 rupees” in future pit-emptying costs if they were to build their own toilet, a large sum for people in these communities. People also appreciated the fact that there was a consistent supply of water at the facility for self-cleaning and flushing, and that helped ensure “that the facility will remain clean.” Finally, the construction of a shared facility helped study participants avoid the issue of building a private toilet on land that they did not own. This facility provided them with a realistic option to end open defecation, one that was enabling them to create a “healthier and cleaner community.”

However, study participants clearly made the point that simply having a shared sanitation facility in the community did not always make it accessible. People noted that users will “go outside” if they have to come from a far distance to reach the facility. This was particularly true for women in the evening. One woman shared that it took her half an hour to reach the facility. This was not a problem during the day but “created a problem at night.” Many of the female study participants described “fear and anxiety” when they had to defecate in the open, especially at night. One female participant recalled having to relieve herself in the middle of the night. She said, “I had to go near the river and definitely felt scared.” If it was raining, they would have to “take an umbrella” and were often surrounded by “scorpions, insects, and disease-causing germs.” Therefore, not having a centrally located shared sanitation facility that is easily accessible throughout the day could force people to revert to open defecation, triggering a host of deleterious psychosocial outcomes.

Overcrowding was sometimes a problem because many users were forced to wait for an open stall. Some would defecate in the open if the wait was too long, indicating that facilities should be designed according to the population of a catchment area. The time constraint was felt acutely by some of the female study participants. They felt the pressure to tend to household chores and could not spend that time waiting in line for a toilet. As such, one woman noted, “whoever has time, for them it is great. But those who don’t have time, it’s a problem.” This was because “women have to look after all the household chores.” As such, participants advocated for more facilities to be built in central locations to improve facility access.

For some, certain design considerations made them feel as though the facility was accessible. For example, at night, “the facility has lights and the facility staff are here, so we come and go comfortably.” Having separate entrances for women and men had a similar effect. These design features alleviated the feeling of “desperation” when they had to defecate in the open, be it “on the side of the road” or in a field, while helping preserve people’s “dignity and respect.” One participant said that “more households have been built here” because people want to live in this community now so that they can access the facility.

**Table 1:** Summary of attributes that constitute a well-managed shared sanitation facility

	Attributes of well-managed shared sanitation facilities	
Maintenance	Provision of water	Helps with flushing, self-cleaning, and facility cleaning
	Professional management	Help ensure functionality, safety, and accountability
	Quality of built infrastructure	Helps encourage use as the facility is something desirable
Access	Short distance from home	Helps ensure that people can walk to the facility day or night
	Short wait time	Helps ensure that people can use the facility in the middle of a busy day
	Design features	Helps people feel safe thereby making the facility accessible

## DISCUSSION

This study had two salient findings. First, our participants noted that maintenance was an essential attribute of managed shared sanitation facilities. In their view, a well-maintained facility had an adequate supply of water, was professionally managed, and was well constructed. On the basis of this finding, we suggest that managed shared facilities be clearly distinguished from unmanaged shared facilities, which are more likely to fall into disrepair and go unused. Second, managed shared sanitation facilities have to be accessible. The distance people have to walk to get to the facility, wait times once they are there, and design features were three factors that contributed to whether a facility was accessible, and thus whether it was high-quality. Having a well-maintained and accessible facility encouraged our participants to use the shared sanitation facility in the community where they lived. As a result, they experienced relief from anxiety and fear, commonly associated with open defecation, and felt that their sense of dignity was being preserved.

Much of what our participants told us confirmed findings from previous studies that highlighted structural barriers to private household toilet ownership. In this study, we found that gender also acted as a barrier to toilet ownership in addition to uncertain land tenure and increased poverty. Women have been found to have less control over household resources and exert less influence on how household resources are allocated. ^26^^–^ ^28^ As such, they might not be able to convince their male counterparts to invest in a toilet, thereby increasing their susceptibility to nonmarital sexual violence and adverse psychosocial outcomes as they are forced to defecate in the open. Yet managed shared sanitation continues to be ignored as a viable option despite these barriers being found in previous research, ^29^^–^ ^32^ and despite global health leaders advocating against one size fits all approaches. ^4^^,^ ^33^^,^ ^34^ Private household toilets remain out of reach for people in our study area, who do not have enough money or land to build one ^35^^,^ ^36^ and who lack the supporting infrastructure, such as waste management and adequate water supply, to make a private toilet feasible. Thus, sanitation policies should address social determinants to actuate the goal of universal coverage of private household toilets. In the meantime, however, providing managed shared sanitation in the interim is akin to an act of “pragmatic solidarity,” one that can help “diminish unjust hardship.” ^24^

In order for shared sanitation to diminish unjust hardship, however, it needs to be of a certain quality, which according to our participants, can be achieved by ensuring maintenance. We suggest that in this context, and similar contexts, managed facilities need to provide water for flushing, self-cleaning, and menstrual hygiene management. ^10^ Water is also required for facility cleaning. Women interviewed in previous studies reported that toilets at unmanaged shared facilities in India were dirty, a fact that discouraged use. ^37^ Another study found that 60% of unmanaged sanitation facilities in their study were dirty if shared by more than 10 households, so a consistent cleaning schedule is especially important as the number of households sharing a toilet facility increases. ^38^ Professional management was a key attribute of well-maintained facilities, according to our participants, who told us that this was associated with a consistent cleaning schedule. The presence of staff could also help ensure safety for women and girls. The emphasis on professional management is important given the backdrop of community participation and management models for water and sanitation utilities in Southeast Asia. ^39^^,^ ^40^ There are myriad issues with these participatory models. For example, committees charged with operating and maintaining these utilities have struggled to recover their costs. ^40^ There are also examples of undemocratic decision-making processes that emerge when caste and gender-based hierarchies are not accounted for before utilities are handed off to communities. ^40^^,^ ^41^ Furthermore, sanitation services require waste management, a task not all communities can handle on their own or without the support of the state or other institutions. ^42^^–^ ^44^ Our participants wanted a professionally run facility in large part so that there would be a system that would ensure that the facility is “connected to a sewer line so that the waste does not flow into the nearby river,” which people use for “bathing and household chores.” Therefore, future research should examine financially sustainable and professional management models of shared sanitation facilities.

Managed shared sanitation must also be accessible. Our participants noted that the distance they had to walk from their home to reach the facility was one measure of accessibility. So was the amount of time they had to wait in line as overdemand for shared toilet facilities can lead to lower user satisfaction. ^45^^,^ ^46^ Women and girls often use toilets for longer periods of time than men and boys while managing menstruation and during pregnancy. As such, more stalls should be provided for women and girls to reduce the amount of time they have to wait to use the toilet at a shared facility. ^47^^,^ ^48^

Other design features, such as the availability of lights at night, help people access the facility after dark. Additionally, shared sanitation facilities can increase their accessibility by ensuring that there are stalls that meet the needs of children, the elderly, and those with physical disabilities. ^49^

This study had two limitations. First, the qualitative nature of this work means that our sample size was small and was restricted to two communities within one district of a state. Although our results may not be generalizable across all states of India, there are likely policy-relevant findings here that can be useful for contextually similar communities that face structural barriers to private toilet ownership. Second, two of the authors are the directors of the organization that manages and operates the facilities where these interviews take place. Given their roles as members of the organization that provides access to shared sanitation in the study communities, we included research collaborators who were not associated with the organization to better ensure that methods and analyses minimized bias.

## CONCLUSION

The Sustainable Development Goals have set an ambitious target to end open defecation by 2030. Managed shared sanitation could help move millions of people up the sanitation ladder away from open defecation. However, unlike private toilets, which are categorized by quality, all forms of shared sanitation are considered “limited” regardless of their quality. This study interviewed shared sanitation users in Jharkhand, India, to elucidate the attributes of well-managed shared sanitation. We found that maintenance and accessibility are key indicators of well-managed shared sanitation. Maintenance includes the provision of water for flushing and self-cleaning, cleaning, and high-quality built infrastructure. Accessibility is defined by the distance people have to walk to reach the facility, the amount of time they had to wait in line, and design features of the facility that encourage use. These findings should help distinguish managed versus unmanaged shared sanitation and could help inform global sanitation policies. Future research should examine other attributes of well managed shared sanitation.
